# The risk of non-steroidal anti-inflammatory drug-induced heart failure in people with chronic kidney disease: a systematic review

**DOI:** 10.1007/s10389-021-01654-3

**Published:** 2021-10-21

**Authors:** Bethany S. Ward, Michael Naughton, Dorothea Nitsch, Mariam Molokhia

**Affiliations:** 1 Department of Population Health Sciences, School of Population Health and Environmental Sciences, Faculty of Life Sciences and Medicine, King’s College London, 3.04, 3rd Floor Addison House, Guy’s Campus, London, SE1 1UL UK; 2 Population Health Sciences, King’s College London, London, UK; 3 Faculty of Epidemiology and Population Health, London School of Hygiene and Tropical Medicine, London, UK

**Keywords:** Anti-inflammatory agents, non-steroidal, Cyclooxygenase 2 inhibitors, Kidney failure, chronic, Heart failure

## Abstract

**Aim:**

To examine the risk of non-steroidal anti-inflammatory drug-induced heart failure in patients with chronic kidney disease.

**Methods:**

Embase, Medline, CENTRAL, Web of Science, and Google Scholar were searched for papers published in English between 1st January 1999 and 31st May 2020. Papers were included if some participants had chronic kidney disease, were exposed to non-steroidal anti-inflammatory drugs, and where heart failure was measured as an outcome. Papers were assessed for risk of bias using the Cochrane Risk of Bias 2 tool for randomised controlled trials, and ROBINS-I for observational studies.

**Results:**

A total of 2480 independent papers were retrieved. Following abstract screening, 165 full texts were reviewed to identify seven eligible papers: two randomised controlled trials, four cohort studies, and one case-control study. For chronic kidney disease (stage 3–5), relative risk for heart failure ranged from 0.3 to 1.9 with 95% confidence interval 0.04 to 15.1. Results were not pooled due to study heterogeneity. We attributed bias to heterogenous populations studied, probable confounding due to partially adjusted risk estimates, and heterogenous measurement of the heart failure outcome.

**Conclusion:**

Overall, there are only a few studies to refute or support an increased risk of heart failure associated with taking non-steroidal anti-inflammatory drugs in patients with chronic kidney disease, and therefore no robust evidence was available.

**Supplementary Information:**

The online version contains supplementary material available at 10.1007/s10389-021-01654-3.

## Introduction

### Background & rationale

Chronic kidney disease (CKD) has a global prevalence of ~11% (including albuminuria), and this figure rises with age (Hill et al. 2016). In England, the lifetime lost health-related quality of life is estimated as £7.18 billion for CKD stages 3–5(Nguyen et al. 2018).

Chronic pain may be present in up to 60% of individuals with CKD (Wu et al. 2015). Older individuals with CKD are more likely to need treatment for pain due to musculoskeletal conditions (Zhang and Rothenbacher 2008). In the UK, the prevalence of chronic pain is estimated to be 44%, increasing to 62% in people over 75 years old (Fayaz et al. 2016). Non-steroidal anti-inflammatory drugs (NSAIDs) such as ibuprofen are widely prescribed to help with joint symptoms of inflammation and pain (Wu et al. 2015); general practice surgeries in England issued 841,738 NSAID prescriptions in January 2021 alone, and 10,219,360 prescriptions throughout 2020 (EBM Data Lab University of Oxford 2020). This may be changing in the future, as a recent NICE guideline on chronic pain has recommended against the use of NSAIDs in chronic primary pain (National Institute for Health and Care Excellence 2021). A systematic review found NSAID use up to 21% in individuals with CKD in Western countries, although use declined with increasing CKD stage (Lefebvre et al. 2019). In general population studies, NSAIDs have been associated with cardiovascular harm, but the evidence for the degree of risk associated with different NSAIDs is poor. NSAIDs are known to cause renal impairment, acute kidney injury, gastrointestinal bleeding, thrombotic events, myocardial infarction, and heart failure (HF) in the general population, which are common complications in CKD. NSAID use is cautioned in people with existing HF or CKD, as CKD itself is associated with an increased risk of developing HF (Zhang et al. 2017).

Much of the evidence of drug harms comes from observational real-world data. Most interventional studies are designed for efficacy, and therefore not powered to investigate harms by level of estimated glomerular filtration rate (eGFR). The literature does not provide robust evidence for the safety of NSAIDs in CKD, as very few studies have exclusively studied individuals with CKD.

A September 2019 search of the Cochrane Central Register of Controlled Trials (CENTRAL) and protocol registry PROSPERO retrieved no systematic reviews looking at the risk of NSAID associated HF in people with CKD, and so a gap in the literature was identified. Therefore, this systematic review was carried out to assess the risk of HF to people with CKD who are exposed to NSAIDs, to inform prescribing choices in CKD.

### Objectives

Our study aim was to assess the risk of HF to a CKD stage 3–5 population of taking NSAIDs. The participants were identified as adults with CKD stage 3–5, i.e., eGFR < 60 ml min^−1^ 1.73 m^−2^. The intervention studied included any single NSAID or combination use and included cyclooxygenase 2 inhibitors (coxibs) and acetylsalicylic acid (aspirin) as well as traditional NSAIDs. Comparison groups could include non-NSAID exposure, placebo, or no treatment. We studied new diagnosis of HF as an outcome.

## Methods

This systematic review has been registered with PROSPERO and can be found with the ID CRD42020192605. Authors have reported according to Preferred Reporting Items for Systematic Reviews and Meta-Analyses(PRISMA) criteria (Moher et al. 2009); the checklist is available as supplementary material. Grading of Recommendations, Assessment, Development, and Evaluations (GRADE) was used to evaluate quality of evidence (Siemieniuk and Guyatt 2021).

### Search strategy

Databases searched included Embase and Medline (via OVID), CENTRAL, Web of Science, Google Scholar, PROSPERO, and Trial registries (International Standard Randomised Controlled Trials Number (ISRCTN), and clinicaltrials.gov), and related conference proceedings and abstracts. The search strategy (see supplementary material): search items used MeSH terms for NSAIDs, kidney disease, and heart failure. For filtering types of study, British Medical Journal Best Practice study design search filters were used to include only randomised controlled trials (RCTs) and observational studies (BMJ 2020). The search was filtered to include studies published between the 1st of January 1999 and the 31st of May 2020.

### Study selection

This review included: (i) randomised controlled trials and observational studies (case–control, case-series, and cohort studies), (ii) in English language, (iii) considering NSAID-exposed participants compared to non-NSAID exposure, no treatment, or placebo, (iv) with CKD ≥ stage 3, ,i,.e., GFR < 60 ml min^−1^ 1.73 m^−2^, and (v) HF as the outcome. HF was diagnosed by New York Heart Association (NYHA) or International Classification of Disease (ICD) criteria or reduced left ventricular ejection fraction (LVEF ≤ 50%). Exclusion criteria were (i) participants < 18 years old, pregnant, or breastfeeding, (ii) case reports, and (iii) pharmacokinetic, pharmacogenetic, or animal studies.

**Fig. 1:**
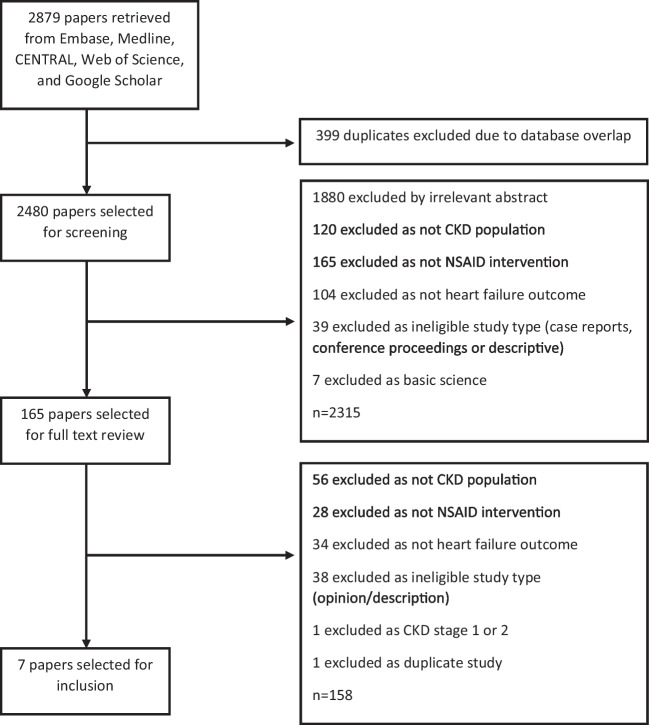
Flow diagram for study selection to find studies looking at the risk of heart failure to participants with chronic kidney disease when exposed to non-steroidal anti-inflammatory drugs

Two authors (BW & MM) screened titles and abstracts identified by the searches. Any disagreements were resolved by consensus discussion; a third reviewer was available (DN) but further adjudication was not required. Relevant full-text papers were retrieved and independently screened by two authors (BW & MM), and any disagreements resolved by consensus. The reasons for exclusion after full-text review were documented [Fig. 1].

### Data extraction

Search results were imported to Endnote for review. Previously specified outcome data was identified in published studies and extracted separately.

Data extracted included: author, publication date, location, study design, population/sampling method, participant inclusion criteria, participant exclusion criteria, NSAID studied, exposure definition, CKD stage/eGFR, unexposed group, sample size, % male/female, outcome, outcome measure, number of cases, confounders adjusted for, reported outcome statistic, outcome as relative risk (with 95% confidence interval), and funding source.

### Quality assessment

Risk of bias assessment was performed on all included studies. RCTs were assessed using the Cochrane Risk of Bias 2 (RoB2) tool (Sterne et al. 2019), and observational studies using ROBINS-I(Sterne et al. 2016). Studies were classified as high, medium, or low risk of bias. Further detail is given in the supplementary material.

A funnel plot was constructed to analyse publication bias within this review using STATA 16.

### Data synthesis and analysis

**Fig. 2:**
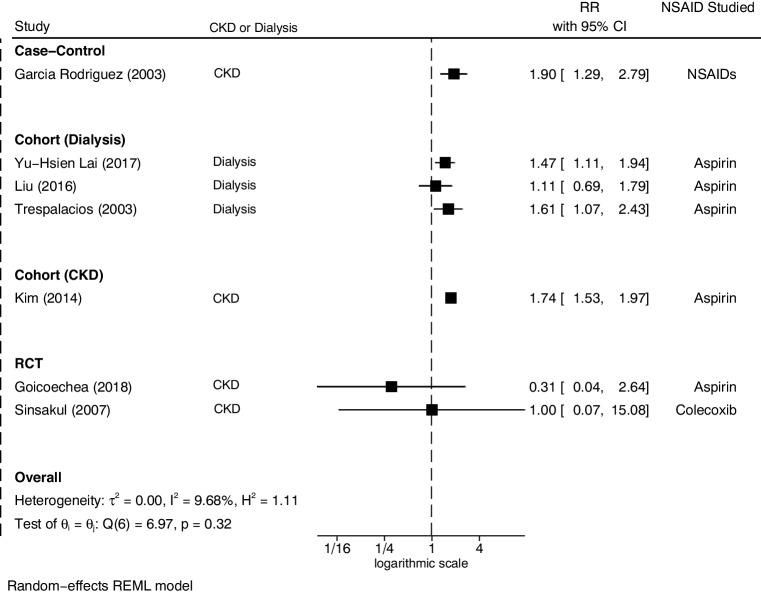
Forest plot of studies looking at the risk of non-steroidal anti-inflammatory drug-induced heart failure in chronic kidney disease, separated by study type alongside relative risk and 95% confidence interval

Individual study relative risks (RR) were investigated by study type (RCT, cohort, and case–control) and displayed in a forest plot including RR with 95% confidence interval (CI) for the appropriate studies [Fig. 2]. For representation in a forest plot, all studies were assigned a RR and 95% CI figure. We used reported adjusted RR from the paper, but those reporting an alternative statistical measure such as hazard ratio (HR) or no measure reported, had a RR and 95% CI calculated based on the original study data.”

STATA 16 software was used for the forest plots, including I^2^ measure for heterogeneity. Random effects models were used to allow for heterogeneity.

Three of the studies looked at solely dialysis patients (CKD stage 5); a subgroup meta-analysis was considered but not deemed appropriate, and risks were reported separately for all studies which included those with moderate to severe CKD (stage 3–4). The GRADE framework was applied to inform recommendations based on the findings of this review.

## Results

### Study selection

A detailed description of study selection is shown in Fig. 1.

Overall, 1676 independent papers were retrieved from Embase and Medline and a further 804 from CENTRAL, Web of Science, Google Scholar, and trial registries (clinicaltrials.gov, and ISRCTN); 2480 independent papers in total. Of these, 2315 papers were excluded by screening the abstracts and titles, removing duplicates and ineligible results based on criteria defined in the protocol. The remaining 165 papers were screened using the full text, leaving seven papers eligible for inclusion.

Cochrane CENTRAL and PROSPERO as well as the database search found no systematic reviews relevant to NSAID-associated HF risks in the CKD population, excluding one which did not find any eligible studies (Marks et al. 2011) and a study which considered the cardioprotective effect of aspirin in CKD with no focus on heart failure or alternative NSAIDs (Qu et al. 2020).

### Study characteristics

Table 1 summarises a description of study characteristics. Studies selected comprised of two RCTs, and five observational studies, consisting of four cohort and one case-control study. Because of the high number of characteristics, it was considered helpful to split them into three separate, vertically-placed groups, each covering different aspects of the seven studies: studies 5 to 7 involved dialysis.

**Table 1:** Study characteristics for papers (1999–2020) included as examining the risk of heart failure in chronic kidney disease when exposed to non-steroidal anti-inflammatory drugs

**Study**	**Location**	**Study Design (Year)**	**Population/ Sampling**	**Inclusion Criteria**	**Exclusion Criteria**		
1. Goicoechea et al. (2018)	Spain	RCT: intervention, open label (2010-2015)	CKD patients- Community of Madrid Centres	eGFR 15–60 ml min^−1^ 1.73 m^−2^ without previous cardiovascular events. Males 45–79 years or females 55–79 years	Previous cardiovascular or vascular disease: arrythmia, arrest, angina, acute MI, stroke, >50% carotid stenosis, documented peripheral vascular arteriopathy. Hospitalisation in the three months prior to inclusion. Acetyl-salicylic acid allergy. Coagulopathy. Thrombocytopenia <150,000 platelets. Liver Disease by hepatitis B or C, or HIV. Immunosuppressive treatment <12 weeks before inclusion		
2. Sinsakul et al. ((2007)	USA	RCT: placebo-controlled, double blinded, crossover (2003-2004)	Diabetic nephropathy patients from North American institutions	Diabetes Mellitus type 1 or 2 and diabetic nephropathy with proteinuria >500 mg d^−1^ or serum creatinine ≤265.2 μmolL^−1^	Patients with other renal diseases, recent history of acute renal failure, hyperkalaemia ≥5.1 mmolL^−1^ at screening, any recent myocardial event, or history of peptic ulcer disease or gastrointestinal bleeding		
3. García Rodríguez and Hernández-Díaz (2003)	UK	Case-Control (1996)	CKD recorded on patient GP records	40-84 years old. Enrolled with GP > 2 years. Hypertension, diabetes, or renal failure in history.	Pregnant, or previous diagnosis of heart failure or cancer		
4. Kim et al. (2014)	South Korea	Retrospective cohort study (1990-2013)	CKD patients at Gachon University Gil Medical Center	CKD eGFR < 60 ml min^−1^ 1.73 m^−2^	<18 years old, records lacking information on serum creatinine levels, and patients who received dialysis prior to entry in the study		
**Dialysis**							
5. Lai et al. (2017)	Taiwan	Cohort (1997-2008)	End stage renal disease on haemodialysis >3 months, with atrial fibrillation and high thromboembolic risk	>3 months on haemodialysis for ESRD; diagnosis of atrial fibrillation, and CHA_2_DS_2_-VASc score >2	Cancer, renal transplant, peritoneal dialysis		
6. Liu et al. (2016)	China	Cohort (2008-2014)	Haemodialysis patients in HD Center of Shanghai No. 1 People's Hospital	18–80 y old, who were undergoing regular haemodialysis for more than 3 months	Active malignancies, haemorrhagic diatheses, liver diseases, platelet counts <100,000/ml, or noncompliant with medical therapy		
7. Trespalacios et al. (2003)	USA	Cohort (1996)	US Renal Data System Dialysis Morbidity and Mortality Wave 2 database (dialysis)	1st date ESRD in 1996, survived dialysis (incl. peritoneal) for 90 days	No pre-existing Congestive Heart Failure		
**Study**	**NSAID studied**	**Exposure Criteria**	**CKD stage/ GFR**	**Unexposed group**	**Sample Size**	**% Female**	**Outcome**
1	Aspirin	Aspirin 100mg daily	3 or 4 -15-60 ml min^−1^ 1.73 m^−2^	CKD patients on standard care - not given aspirin	111 in total, 50 aspirin, 61 non-aspirin	32.4% overall, 36% aspirin 29.5% non-aspirin	Congestive heart failure
2	Celecoxib	Celecoxib 200mg daily for 6 weeks	3 -mean 41.9 ml min^−1^ 1.73 m^−2^	Placebo	24	Not Reported	Cardiac event/ congestive heart failure
3	NSAIDs: aceclofenac, acemetacin, diclofenac, etodolac, fenbufen, fenoprofen, flurbiprofen, ibuprofen, indomethacin, ketoprofen, mefenamic acid and tiaprofenic acid.	Prescription within last month	‘renal failure’ listed on GP record	Non-users of NSAIDs	2278 (46 with renal failure)	48% in whole study. Subgroup not reported.	Heart Failure
4	Aspirin	Aspirin 100mg daily	Stage eGFR < 60 ml min^−1^ 1.73 m^−2^	Non-user of aspirin	2751 in total, 1380 aspirin, 1371 non-aspirin	41.3% in aspirin, 51.8% in non-aspirin	Cardiovascular disease
**Dialysis**							
5	Aspirin	Not Reported	5 / End Stage Renal Disease ICD9 code 585 which states GFR <15 ml min^−1^ 1.73 m^−2^	Not taking aspirin	951 in total, 814 aspirin and 137 none	62% overall, 60% in aspirin, 75% non-aspirin	Congestive Heart Failure
6	Aspirin	Aspirin prescription in records - 100mg daily	Diagnosis of one of: Hypertensive renal injury, Diabetic kidney disease, Chronic glomerulonephritis, Biopsy-proven IgAN, Polycystic kidney disease, Gout kidney, Obstructive nephropathy, Lupus nephritis, Chronic interstitial nephritis, Postrenal transplantation	No aspirin prescription	406 in total, 152 aspirin, 254 non-aspirin	53%	Congestive Heart Failure
7	Aspirin	Not Reported	End stage renal disease patients so assumed Stage 5	Not taking aspirin	436	Not Reported	Hospitalisation for Congestive Heart Failure
**Study**	**Outcome Measure**	**Number of Cases**	**Confounders Adjusted for**	**Reported statistic**	**RR (95% CI)**	**Comments**	**Funding**
1	Congestive heart failure= LVEF <40% and congestive symptoms classified by the New York Heart Association as II to IV	5/50 in ASA, 17/61 in standard.	Assumed not adjusted	HR 0.40 (0.15–1.08)	0.31 (0.04-2.64)	May include dialysis; composite outcome	Sociedad Española de Nefrología (SEN) and Sociedad Madrileña de Nefrología (SOMANE). MG, GFJ, DA, AO, and JL are supported by ISCIII RETIC REDINREN RD016/009 and FEDER funds.
2	Not Reported	1 in each group	Treatment, baseline protein excretion, study site, period, systolic blood pressure, weight, and interaction between treatment and period	None	1.00 (0.07-15.08)	May include dialysis; all diabetic; composite outcome	Support from National Institutes of Health and Pfizer
3	GP reported dyspnoea, with pulmonary oedema; peripheral oedema and raised jugular venous pressure on examination; or evidence of heart disease	1780	Age, sex, BMI, smoking, alcohol use, comedication and comorbidity	RR 1.9 (1.3-2.8)	1.9 (1.3-2.8)	May include dialysis; not all participants CKD	Research grant from AstraZeneca (pharmacological company)
4	angina pectoris, myocardial infarction, other ischemic heart disease, atrial fibrillation, heart failure, or cerebrovascular disease on electronic records	490 in aspirin, 279 in non-aspirin	age, gender, comorbidities, proteinuria, baseline eGFR, baseline bloods, and medications.	HR 2.106 (1.82-2.44)	1.74 (1.54-1.98)	No dialysis, some participants with previous cardiovascular disease.	Chong Kun Dang Pharmaceutical Corp. in Korea
**Dialysis**							
5	ICD-9 codes: 398.91, 402.X1, 404.X1, 404.X3, 422, 425, or 428	341 in aspirin, 39 in no treatment	Age, sex, diabetes mellitus, hypertension, and hyperlipidaemia	HR 1.28 (0.91-1.81)	1.47 (1.11-1.94)	Haemodialysis only	Grant from Tzu Chi General Hospital
6	New York Heart Failure Classification	60 cases of CHF diagnosis, 24 aspirin/36 non-aspirin	Mortality figures adjusted for age, sex, Hb, albumin, type 2 diabetes, cancer, and ultrafiltration per week	RR 1.11 (0.69–1.79)	1.11 (0.69–1.79)	Haemodialysis; mixed renal problems	Grants from Shanghai medical key subject construction project, Key Discipline Construction Project of Pudong Health Bureau of Shanghai, Outstanding Leaders Training Programme of the Pudong Health Bureau of Shanghai, and Fudan University Advantages of Disciplines Building
7	Hospitalised in 'Medicare' and CHF from ICD9 code 428.x	27	Age, sex, diabetes, previous coronary heart disease, and haematocrit	HR:1.59 (1.01-2.50)	1.61 (1.07-2.43)	All dialysis; hospitalised with CHF	United States of America Government

### HF risks in CKD stages 3 & 4

García Rodríguez et al. reported the highest RR for HF, in a mixed population of hypertensive, diabetic, and CKD participants (defined as individuals with ‘renal failure’ recorded on their general practice records encompassing individuals with CKD), and included several different NSAIDs (García Rodríguez and Hernández-Díaz 2003). The population limits any conclusions which might be drawn relating to CKD, because individuals with hypertension and diabetes (without CKD) were also included and were at high risk of HF. The Kim et al. cohort study shows an increased risk of all cardiovascular events for CKD patients not using dialysis (eGFR < 60 ml min^−1^ 1.73 m^−2^) when prescribed aspirin, although no specific risk for HF was estimated (Kim et al. 2014).

The two randomised controlled trials examining NSAID use in CKD stages 3 or 4 do not suggest increased risks of taking NSAIDs (aspirin/celecoxib) on the development of HF or cardiac events (Goicoechea et al. 2018; Sinsakul et al. 2007). However, numbers are small and a composite outcome was used which may allow cardioprotective effects to counteract the HF risk. Sinsakul et al. included an exclusively diabetic population with CKD (diabetic nephropathy with proteinuria > 500 mg d ^− 1^ or serum creatinine ≤ 265.2 μmol L^−1^), thus limiting generalisability (Sinsakul et al. 2007). A composite outcome was used, ‘cardiac event/congestive heart failure’, so HF cannot be specifically identified. The small sample size (24 participants) means the group may not be representative of the wider population, and this increases the likelihood of chance or outliers affecting the result, demonstrated in the wide 95% CI of 0.07–15.08. The Goicoechea et al. study was a highly selective trial of patients with eGFR 15–60 ml min^−1^ 1.73 m^−2^ which excluded participants with co-morbidities, exposed participants to low-dose aspirin, as indicated for primary prevention of cardiovascular disease, and also studied a small sample of 111 participants (Goicoechea et al. 2018). The low dose may be the reason for the reported protective effect of aspirin in this study, but the lack of serious comorbidities may also be a contributing factor.

We were unable to estimate pooled risks for stages 3 and 4 of CKD, as studies found were based on small numbers of individuals with CKD and included composite outcomes or populations, deemed not suitable for inclusion in a meta-analysis. Figure 2 shows a forest plot to summarise the results of each study as RR and 95% CI.

### HF risks in renal dialysis CKD (stage 5)

This review could suggest participants undergoing dialysis (stage 5 CKD) have increased risk of HF when prescribed aspirin. If analysed assuming suitable homogeneity, the combined RR for the studies containing participants on dialysis is 1.43 (95% CI 1.16–1.76). However the dialysis participants in two of the studies had significant underlying CVD (Lai et al. 2017; Trespalacios et al. 2003), although lower in Liu et al. (2016), suggesting these results are likely to be confounded by underlying CVD.

### Risk of bias within studies

We undertook a GRADE assessment (Siemieniuk and Guyatt 2021) to examine bias in the meta-analysis results, which can be found in full in the supplementary material. We found no firm evidence to suggest an increased risk of aspirin-associated HF in dialysis patients (CKD stage 5), due to probable confounding by indication (i.e., patients were likely to be prescribed aspirin for underlying IHD, which is a known risk for HF).

Both RCTs were found to be of medium risk of bias overall using the Cochrane RoB2 tool (Table 2). All four observational studies received medium risk of bias judgements using the ROBINS-I tool (Table 3).

**Table 2:** Cochrane Risk of Bias 2 tool (Sterne et al. 2019) for randomised controlled trials

Study (Cochrane Risk of Bias 2 tool)	Randomisation	Assignment	Adherence	Missing outcome data	Measurement of the outcome	Selection of reported results	Overall
Goicoechea et al. (2018)	medium	medium	medium	low	medium	medium	medium
Sinsakul et al. (2007)	low	medium	low	low	medium	high	medium

**Table 3:** Cochrane ROBINS-I(Sterne et al. 2016) tool for observational study bias

Study	Confounding	Selection of participants	Classification of interventions	Deviation from intended intervention	Missing data	Measurement of outcome	Selection of reported result	Overall
Cohort								
Lai et al. (2017)	moderate	low	low	low	moderate	moderate	moderate	moderate
Liu et al. (2016)	moderate	low	low	low	low	moderate	moderate	moderate
Trespalacios et al. (2003)	moderate	moderate	low	low	moderate	moderate	low	moderate
Kim et al. (2014)	moderate	moderate	low	low	moderate	moderate	low	moderate
Case–control								
García Rodríguez et al. (2003)	moderate	serious	low	low	moderate	moderate	low	moderate

Further detail on the risk of bias assessment can be found in the supplementary material.

The supplementary material also contains a funnel plot aiming to indicate the risk of publication bias. The small number of studies makes it difficult to draw conclusions, but the plot is largely symmetrical around the mean effect size, and standard error is small in several of the studies.

## Discussion

### Summary

We were unable to estimate NSAID-induced HF risks separately for CKD stages 3, 4, or 5 due to insufficient data.

Lai et al. demonstrated the risk of congestive HF to a CKD stage 5 haemodialysis population with high thromboembolic risk and atrial fibrillation when prescribed aspirin. The study focussed on a subset of participants with atrial fibrillation, who are known to have increased risks of HF, probably due to underlying ischaemic heart disease.

Liu et al. studied a haemodialysis population with heterogenous kidney disease including hypertensive renal injury, diabetic kidney disease, polycystic kidney disease, lupus nephritis, and chronic interstitial nephritis. Although the participants had a lower prevalence of cardiovascular disease co-morbidity (9.2%) than the other studies included, this may still cause biased results due to increased risks of HF.

Trespalacios et al. (2003) undertook a retrospective cohort study using the US Renal Data System Dialysis Morbidity and Mortality Wave 2 database for chronic dialysis patients (both haemodialysis and peritoneal) to assess their risk of hospitalisation for HF. Trespalacios et al. (2003) noted a large proportion of pre-existing cardiovascular disease (30.2% in HF hospitalisation cases and 23.4% in those not hospitalised for HF) and prevalent hypertension. Around a quarter of participants were African American, and the use of only ‘Medicare’ participants limits the study to a largely older population. All these factors are likely to affect results through selection bias and confounding.

### Comparison with existing literature

This review considered HF risk to CKD patients partially due to the established evidence for NSAID- associated HF in the general population. A meta-analysis of over 700 randomised trials in the general population, which included people with CKD, discovered that all the NSAIDs studied led to a doubled risk of HF diagnosis or admissions (Coxib and traditional NSAID Trialists’ (CNT) Collaboration 2013). Most of the studies included in this review studied aspirin, which may confer lower risks of HF than other NSAIDs.

A Cochrane systematic review intended to review RCT evidence of safety of NSAIDs (amongst other pain medications) in a population with rheumatoid arthritis and comorbid renal conditions (Marks et al. 2011). No studies were found eligible for inclusion in this review, highlighting the lack of trials looking at NSAID safety in individuals with CKD, with or without comorbidities. However, a recent systematic review found that aspirin had no cardioprotective effects in the CKD population, with no significant reduction in stroke, MI, heart failure, or cardiovascular disease (Qu et al. 2020). Qu et al. included data from three studies (Kim et al. 2014; Lai et al. 2017; and Goicoechea et al. 2018) found eligible for this review (Qu et al. 2020). Well-designed randomised trials are needed to clarify the risk of HF when prescribing NSAIDs to the CKD population. A randomised controlled trial, 'ATTACK' (aspirin to target arterial events in chronic kidney disease), is currently investigating low-dose aspirin in CKD for prevention of major vascular events; results are awaited and will contribute to the evidence base (National Institute for Health Research 2018).

Iwagami et al. (2018) showed that HF and acute kidney injury were the top cause-specific hospital admission outcomes in those with CKD stages 3–5 compared to age- and sex-matched controls (Iwagami et al. 2018). HF prevalence increases with age from around 1% in a general population aged 55–64 to 17.4% in those aged ≥ 85 (Levy et al. 2002). Previous studies, based in the United States, have estimated that individuals with CKD have a 3-fold increased risk of incident HF (Kottgen et al. 2007).

NSAIDs provide an analgesic and anti-inflammatory effect but have also been used for cardiovascular protection; aspirin is prescribed in low doses to people with high risk of a thrombotic event such as a stroke or myocardial infarction (García Rodríguez and Hernández-Díaz 2003). NSAIDs, including aspirin, are best used when benefits to reduction of cardiovascular disease are balanced against risk of bleeding, so it is important to assess risks of HF in dialysis patients when deciding on medications. Additionally, it is difficult to produce research comparing NSAIDs to no treatment due to ethical concerns regarding pain treatment.

### Strengths & limitations

This is a comprehensive systematic review on an important clinical problem. The search of literature was thorough, and relevant papers have been discussed. This review has not attempted to run an innappropriate meta-analysis using a small group of heterogenous studies, and instead highlights the necesity for a stronger and more CKD-focussed evidence base.

Unfortunately, there are several possible sources of bias within the sources reviewed, including study population, confounding by indication through underlying CHD, NSAID exposure, and HF classification.

The García Rodríguez et al. (2003) high RR was not attributable to CKD participants alone, as they were analysed as one population with hypertensive and diabetic patients, with 2% of the group categorised as having ‘renal failure’, a term previously used to describe CKD but also established renal failure requiring dialysis. García Rodríguez et al. reported on NSAID safety in a diverse, high-risk population, but do not provide sufficient evidence to study effects in an exclusively CKD population. Heterogenous populations were included, with varying definitions of CKD.

There is possible misclassification of exposure to NSAIDs due to over-the-counter use. We were unable to ascertain participant adherence to NSAID prescriptions and the correct doses were assumed, which could underestimate the true risk. Incomplete adjusting for other comorbidities and confounders, including deprivation, may cause further bias in estimates.

Kim et al. (2014) used cardiovascular events as an endpoint, and reported no separate figures of HF incidence.

A possible reason for the differences in the studies on general CKD is that a variety of NSAIDs were considered the exposure. Arfè et al. (2016) demonstrated significant differences between NSAIDs in relation to risk of hospitalisation for HF, reporting celecoxib odds ratio (OR) = 0.96 (95% CI 0.90–1.02) whereas ibuprofen OR = 1.24 (95% CI 1.07–1.43) and ketorolac was found to have OR = 1.85 (95% CI 1.62–2.12). It would be important to know the risk associated with each NSAID for people with CKD, as it is likely that risk also varies between NSAIDs in the CKD population.

Finally, it is possible that some of these results may have been due to chance.

## Conclusion

### Key findings

There is no firm evidence for NSAID-induced HF in CKD, and consequently more work is needed. We were unable to estimate HF risk in CKD separately for stages 3, 4, or 5.

### Implications for research and practice

The clinical relevance of this review is focussed in the dialysis studies, looking at the most severely affected patients (CKD stage 5) but does not suggest HF may be attributable to aspirin, due to confounding through underlying CHD. The evidence for CKD stages 3 and 4 is inconclusive, as studies were small and prone to bias. Well-conducted larger studies are required to determine risk of HF associated with NSAIDs in CKD Stage 3 and 4 and inform future guidelines and practice. If NSAIDS are used in CKD populations, risks for HF, bleeding, acute kidney injury, and other adverse effects should be carefully monitored and mitigated against (Zhang et al. 2017). A balanced risk–benefit analyis can help inform clinicians and patients to enable the best decisions for care.

## Supplementary Information

ESM 1 (PDF 570 kb)

ESM 2 (PNG 26873 kb)

High resolution image (TIF 1549 kb)

ESM 3 (PDF 115 kb)

ESM 4 (PDF 119 kb)

ESM 5 (PDF 200 kb)

## Data Availability

The data underlying this article will be shared on reasonable request to the corresponding author.
