# Supplementary material for: The risk of non-steroidal anti-inflammatory drug-induced heart failure in people with chronic kidney disease: a systematic review
Source: J Public Health (Berl). 2021 Oct 21;30(7):1763–73. doi: 10.1007/s10389-021-01654-3 (PMC12380885; doi:10.1007/s10389-021-01654-3)
Supplement: Supplementary file 1 — (PDF 570 kb) [file 10389_2021_1654_MOESM1_ESM.pdf]

Supplementary Information Figure 1: Full Search Strategy

Embase full search strategy

1. exp Nonsteroid Antiinflammatory Agent/
2. (non steroid* antiinflammatory agent* or non steroid* anti inflammatory agent*).tw.
3. nsaid*.tw.
4. exp Cyclooxygenase 2 Inhibitor/
5. exp heart failure/
6. exp chronic kidney disease/
7. (ACECLOFENAC or ASPIRIN or "BENZYDAMINE HYDROCHLORIDE" or BROMFENAC or CELECOXIB or DEXIBUPROFEN or DEXKETOPROFEN or DICLOFENAC or ETODOLAC or ETORICOXIB or FELBINAC or FLURBIPROFEN or IBUPROFEN or INDOMETACIN or KETOPROFEN or "KETOROLAC TROMETAMOL" or "MEFENAMIC ACID" or MELOXICAM or NABUMETONE or NAPROXEN or NEPAFENAC or PARECOXIB or PIROXICAM or SULINDAC or TENOXICAM or TIAPROFENIC ACID or "TOLFENAMIC ACID").mp.
8. reduced LVEF.mp. [mp=title, abstract, original title, name of substance word, subject heading word, floating sub-heading word, keyword heading word, organism supplementary concept word, protocol supplementary concept word, rare disease supplementary concept word, unique identifier, synonyms]
9. "reduced left ventricular ejection fraction".mp. [mp=title, abstract, original title, name of substance word, subject heading word, floating sub-heading word, keyword heading word, organism supplementary concept word, protocol supplementary concept word, rare disease supplementary concept word, unique identifier, synonyms]
10. cardiac failure.mp. [mp=title, abstract, original title, name of substance word, subject heading word, floating sub-heading word, keyword heading word, organism supplementary concept word, protocol supplementary concept word, rare disease supplementary concept word, unique identifier, synonyms]
11. chronic kidney disease.mp. [mp=title, abstract, original title, name of substance word, subject heading word, floating sub-heading word, keyword heading word, organism supplementary concept word, protocol supplementary concept word, rare disease supplementary concept word, unique identifier, synonyms]
12. (Chronic and (renal or kidney)).mp. and (fail* or insuff*).tw. [mp=title, abstract, original title, name of substance word, subject heading word, floating sub-heading word, keyword heading word, organism supplementary concept word, protocol supplementary concept word, rare disease supplementary concept word, unique identifier, synonyms]
13. end stage renal.mp. [mp=title, abstract, original title, name of substance word, subject heading word, floating sub-heading word, keyword heading word, organism supplementary concept word, protocol supplementary concept word, rare disease supplementary concept word, unique identifier, synonyms]
14. (Chronic and (renal or kidney) and (fail* or insuff*)).mp. [mp=title, abstract, original title, name of substance word, subject heading word, floating sub-heading word, keyword

heading word, organism supplementary concept word, protocol supplementary concept word, rare disease supplementary concept word, unique identifier, synonyms]

15. (CKD or CKF or CKI or CRD or CRF or CRI).mp. [mp=title, abstract, original title, name of substance word, subject heading word, floating sub-heading word, keyword heading word, organism supplementary concept word, protocol supplementary concept word, rare disease supplementary concept word, unique identifier, synonyms]

16. cyclo?oxygenase 2 inhibitor.mp. [mp=title, abstract, original title, name of substance word, subject heading word, floating sub-heading word, keyword heading word, organism supplementary concept word, protocol supplementary concept word, rare disease supplementary concept word, unique identifier, synonyms]

17. kidney disease*.mp. [mp=title, abstract, original title, name of substance word, subject heading word, floating sub-heading word, keyword heading word, organism supplementary concept word, protocol supplementary concept word, rare disease supplementary concept word, unique identifier, synonyms]

18. kidney fail*.mp. [mp=title, abstract, original title, name of substance word, subject heading word, floating sub-heading word, keyword heading word, organism supplementary concept word, protocol supplementary concept word, rare disease supplementary concept word, unique identifier, synonyms]

19. kidney insuff*.mp. [mp=title, abstract, original title, name of substance word, subject heading word, floating sub-heading word, keyword heading word, organism supplementary concept word, protocol supplementary concept word, rare disease supplementary concept word, unique identifier, synonyms]

20. renal fail*.mp. [mp=title, abstract, original title, name of substance word, subject heading word, floating sub-heading word, keyword heading word, organism supplementary concept word, protocol supplementary concept word, rare disease supplementary concept word, unique identifier, synonyms]

21. renal disease*.mp. [mp=title, abstract, original title, name of substance word, subject heading word, floating sub-heading word, keyword heading word, organism supplementary concept word, protocol supplementary concept word, rare disease supplementary concept word, unique identifier, synonyms]

22. renal insuff*.mp. [mp=title, abstract, original title, name of substance word, subject heading word, floating sub-heading word, keyword heading word, organism supplementary concept word, protocol supplementary concept word, rare disease supplementary concept word, unique identifier, synonyms]

23. exp review/

24. (literature adj3 review$).ti,ab.

25. exp meta analysis/

26. exp Systematic Review/

27. 23 or 24 or 25 or 26

28. (medline or medlars or embase or pubmed or cinahl or amed or psychlit or psyclit or psychinfo or psycinfo or scisearch or cochrane).ti,ab.

29. (systematic$ adj2 (review$ or overview)).ti,ab.

30. (meta?anal$ or meta anal$ or meta-anal$ or metaanal$ or metanal$).ti,ab.

31. (random$ or placebo$ or single blind$ or double blind$ or triple blind$).ti,ab.
32. (animal$ not human$).sh,hw.
33. (book or conference paper or editorial or letter or review).pt. not exp randomized controlled trial/
34. (random sampl$ or random digit$ or random effect$ or random survey or random regression).ti,ab. not exp randomized controlled trial/
35. exp cohort analysis/
36. exp longitudinal study/
37. exp prospective study/
38. exp follow up/
39. cohort$.tw.
40. exp case control study/
41. (case$ and control$).tw.
42. exp case study/
43. (case$ and series).tw.
44. 35 or 36 or 37 or 38 or 39 or 40 or 41 or 42 or 43
45. heart failure.mp. [mp=title, abstract, original title, name of substance word, subject heading word, floating sub-heading word, keyword heading word, organism supplementary concept word, protocol supplementary concept word, rare disease supplementary concept word, unique identifier, synonyms]
46. kidney disease/
47. 6 or 11 or 12 or 13 or 14 or 15 or 17 or 18 or 19 or 20 or 21 or 22 or 46
48. 5 or 8 or 9 or 10 or 45
49. 1 or 2 or 3 or 4 or 7 or 16
50. 47 and 48 and 49
51. 31 not (32 or 33 or 34)
52. 27 and 28
53. 52 or 29 or 30
54. 44 or 51 or 53
55. 50 and 54
56. 55
57. limit 56 to (human and english language and yr="1999-2020")

Medline full search strategy

1. Kidney Diseases/
2. Kidney Failure, Chronic/
3. Kidney Failure/
4. renal replacement therapy/ or exp renal dialysis/ or exp hemofiltration/

5. (chronic kidney or chronic renal).tw.

6. (CKD or CKF or CRD or CRF or ESKD or ESRD or ESKF or ESRF).tw.

7. (predialysis or dialysis).tw.

8. (haemodialysis or haemodialysis).tw.

9. (CAPD or CCPD or APD).tw.

10. 1 or 2 or 3 or 4 or 5 or 6 or 7 or 8 or 9

11. exp Heart Failure/

12. ((heart or cardiac or myocard*) adj2 (fail* or insufficien* or decomp*)).tw.

13. 11 or 12

14. (ACECLOFENAC or ASPIRIN or "BENZYDAMINE HYDROCHLORIDE" or BROMFENAC or CELECOXIB or DEXIBUPROFEN or DEXKETOPROFEN or DICLOFENAC or ETODOLAC or ETORICOXIB or FELBINAC or FLURBIPROFEN or IBUPROFEN or INDOMETACIN or KETOPROFEN or "KETOROLAC TROMETAMOL" or "MEFENAMIC ACID" or MELOXICAM or NABUMETONE or NAPROXEN or NEPAFENAC or PARECOXIB or PIROXICAM or SULINDAC or TENOXICAM or TIAPROFENIC ACID or "TOLFENAMIC ACID").mp.

15. exp Anti-Inflammatory Agents, Non-Steroidal/

16. nsaid*.mp. [mp=title, abstract, original title, name of substance word, subject heading word, floating sub-heading word, keyword heading word, organism supplementary concept word, protocol supplementary concept word, rare disease supplementary concept word, unique identifier, synonyms]

17. (non steroid* adj2 anti?inflammator*).mp. [mp=title, abstract, original title, name of substance word, subject heading word, floating sub-heading word, keyword heading word, organism supplementary concept word, protocol supplementary concept word, rare disease supplementary concept word, unique identifier, synonyms]

18. cyclo?oxygenase 2 inhibitor.mp. [mp=title, abstract, original title, name of substance word, subject heading word, floating sub-heading word, keyword heading word, organism supplementary concept word, protocol supplementary concept word, rare disease supplementary concept word, unique identifier, synonyms]

19. exp Cyclooxygenase 2 Inhibitors/

20. 14 or 15 or 16 or 17 or 18 or 19

21. end stage renal.mp. [mp=title, abstract, original title, name of substance word, subject heading word, floating sub-heading word, keyword heading word, organism supplementary concept word, protocol supplementary concept word, rare disease supplementary concept word, unique identifier, synonyms]

22. exp Kidney Diseases/

23. ((kidney or renal) adj2 (fail* or insuff* or disease*)).mp. [mp=title, abstract, original title, name of substance word, subject heading word, floating sub-heading word, keyword heading word, organism supplementary concept word, protocol supplementary concept word, rare disease supplementary concept word, unique identifier, synonyms]

24. 10 or 21 or 22 or 23

25. reduced LVEF.mp. [mp=title, abstract, original title, name of substance word, subject heading word, floating sub-heading word, keyword heading word, organism

supplementary concept word, protocol supplementary concept word, rare disease supplementary concept word, unique identifier, synonyms]

26. (reduced and "left ventricular ejection fraction").mp. [mp=title, abstract, original title, name of substance word, subject heading word, floating sub-heading word, keyword heading word, organism supplementary concept word, protocol supplementary concept word, rare disease supplementary concept word, unique identifier, synonyms]
27. 13 or 25 or 26
28. 20 and 24 and 27
29. review.pt.
30. (medline or medlars or embase or pubmed or cochrane).tw,sh.
31. (scisearch or psychinfo or psycinfo).tw,sh.
32. (psychlit or psyclit).tw,sh.
33. cinahl.tw,sh.
34. ((hand adj2 search$) or (manual$ adj2 search$)).tw,sh.
35. (electronic database$ or bibliographic database$ or computeri?ed database$ or online database$).tw,sh.
36. (pooling or pooled or mantel haenszel).tw,sh.
37. (peto or dersimonian or der simonian or fixed effect).tw,sh.
38. (retraction of publication or retracted publication).pt.
39. 30 or 31 or 32 or 33 or 34 or 35 or 36 or 37 or 38
40. 29 and 39
41. meta-analysis.pt.
42. meta-analysis.sh.
43. (meta-analys$ or meta analys$ or metaanalys$).tw,sh.
44. (systematic$ adj5 review$).tw,sh.
45. (systematic$ adj5 overview$).tw,sh.
46. (quantitativ$ adj5 review$).tw,sh.
47. (quantitativ$ adj5 overview$).tw,sh.
48. (quantitativ$ adj5 synthesis$).tw,sh.
49. (methodologic$ adj5 review$).tw,sh.
50. (methodologic$ adj5 overview$).tw,sh.
51. (integrative research review$ or research integration).tw.
52. 41 or 42 or 43 or 44 or 45 or 46 or 47 or 48 or 49 or 50 or 51
53. 40 or 52
54. randomized controlled trial.pt.
55. (random$ or placebo$ or single blind$ or double blind$ or triple blind$).ti,ab.
56. (retraction of publication or retracted publication).pt.
57. 54 or 55 or 56
58. (animals not humans).sh.

59. ((comment or editorial or meta-analysis or practice-guideline or review or letter) not randomized controlled trial).pt.

60. (random sampl$ or random digit$ or random effect$ or random survey or random regression).ti,ab. not randomized controlled trial.pt.

61. 57 not (58 or 59 or 60)

62. exp cohort studies/

63. cohort$.tw.

64. controlled clinical trial.pt.

65. epidemiologic methods/

66. limit 65 to yr=1966-1989

67. exp case-control studies/

68. (case$ and control$).tw.

69. (case$ and series).tw.

70. 62 or 63 or 64 or 66 or 67 or 68 or 69

71. 53 or 61 or 70

72. 28 and 71

73. 72

74. limit 73 to (english language and humans and yr="1999-2020")

Adapted for CENTRAL, Web of Science, and Google Scholar

("nonsteroid* antiinflammatory agent" OR "non steroid* antiinflammatory agent*" OR "non steroid* anti inflammatory agent*" OR "non?steroidal anti?inflammatory" OR nsaid* OR "cyclooxygenase 2 inhibitor" OR aceclofenac OR aspirin OR "benzydamine hydrochloride" OR bromfenac OR celecoxib OR dexibuprofen OR dexketoprofen OR diclofenac OR etodolac OR etoricoxib OR felbinac OR flurbiprofen OR ibuprofen OR Indometacin OR ketoprofen OR "ketorolac trometamol" OR "mefenamic acid" OR meloxicam OR nabumetone OR naproxen OR nepafenac OR parecoxib OR sulindac OR tenoxicam OR "tiaprofenic acid" OR "tolfenamic acid" OR "cyclo?oxygenase 2 inhibitor) AND ("heart failure" OR "reduced LVEF" OR "reduced left ventricular ejection fraction" OR "cardiac failure") AND ("chronic kidney disease" OR (chronic AND (renal OR kidney) AND (fail* OR insuff*)) OR "end?stage renal" OR CKD OR CKF

OR CKIOR CRD ORCRF OR CRI OR "kidney disease" OR ((kidney OR renal) AND (fail* OR insuff*)) OR "renal disease" OR haemodialysis OR dialysis OR hemodialysis) AND (("cohort analysis" OR "longitudinal study" OR "prospective study" OR "follow up" OR cohort* OR "case control study" OR (case* AND control*) OR "case study" OR (case* AND series*) OR (random* OR placebo* OR "single blind*" OR "double blind*" OR "tripple blind*") OR (review OR "meta analysis" OR "systematic review" AND (medline OR medlars OR embase OR pubmed OR cinahl OR amed OR psychlit OR psyclit OR psychinfor OR scisearch OR cochrane)) OR (systematic* adj2 (review* OR overview)) OR (meta?anal* OR "meta anal*" OR meta-anal* OR metaanal* OR metanal*))

"chronic kidney insufficiency" OR "chronic kidney disease" OR "kidney disease" OR "kidney failure" OR "renal failure" OR "renal insufficiency" OR "dialysis" OR "haemodialysis"

AND

"heart failure" OR "cardiac failure" OR "reduced LVEF" OR "reduced left ventricular ejection fraction"

AND

"NSAID" OR "NSAIDS" OR "non-steroidal anti-inflammatory" OR "nonsteroidal anti-inflammatory" OR "non-steroidal antiinflammatory" OR "nonsteroidal antiinflammatory" OR "cyclo-oxygenase 2 inhibitor" OR "aceclofenac" OR "aspirin" OR "benzydamine hydrochloride" OR "bromfenac" OR "celecoxib" OR "dexibuprofen" OR "dexketoprofen" OR "diclofenac" OR "etodolac" OR "etoricoxib" OR "felbinac" OR "flurbiprofen" OR "ibuprofen" OR "indometacin" OR "ketoprofen" OR "ketorolac trometamol" OR "mefenamic acid" OR "meloxicam" OR "nabumetone" OR "naproxen" OR "nepafenac" OR "parecoxib" OR "sulindac" OR "tenoxicam" OR "tiaprofenic acid" OR "tolfenamic acid" OR "cyclooxygenase 2 inhibitor"

AND

"cohort analysis" OR "longitudinal study" OR "prospective study" OR "follow up" OR cohort* OR "case control study" OR case* control* OR "case study" OR case* series* OR random* OR placebo* OR "single blind*" OR "double blind*" OR "tripple blind*" OR review OR "meta analysis" OR "systematic review" OR systematic* adj2 review* OR overview OR meta?anal* OR "meta anal*" OR meta-anal* OR metaanal* OR metanal*
