# Supplementary figures and images for: The risk of non-steroidal anti-inflammatory drug-induced heart failure in people with chronic kidney disease: a systematic review
Source: J Public Health (Berl). 2021 Oct 21;30(7):1763–73. doi: 10.1007/s10389-021-01654-3 (PMC12380885; doi:10.1007/s10389-021-01654-3)

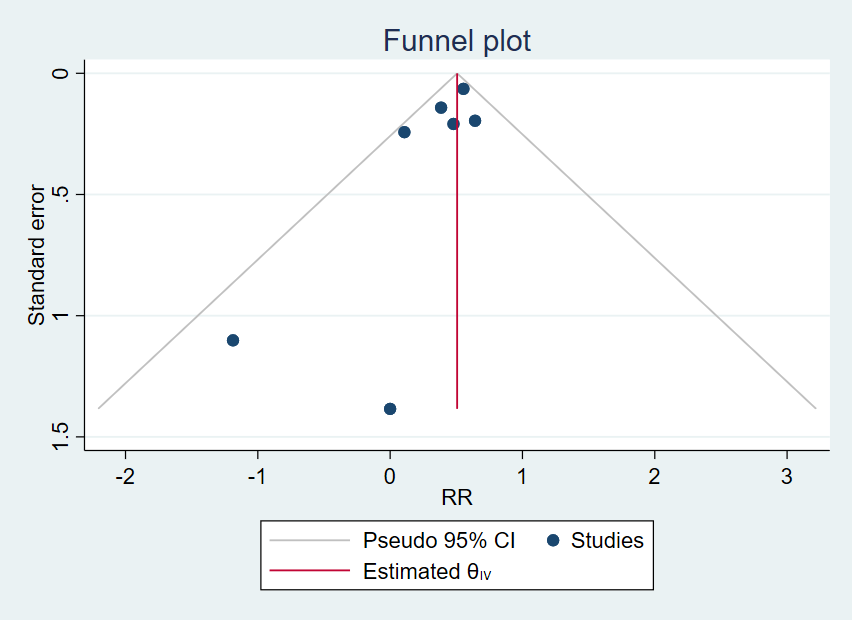

Supplement: Supplementary file 2 — (PNG 26873 kb) [file 10389_2021_1654_Fig3_ESM.png]

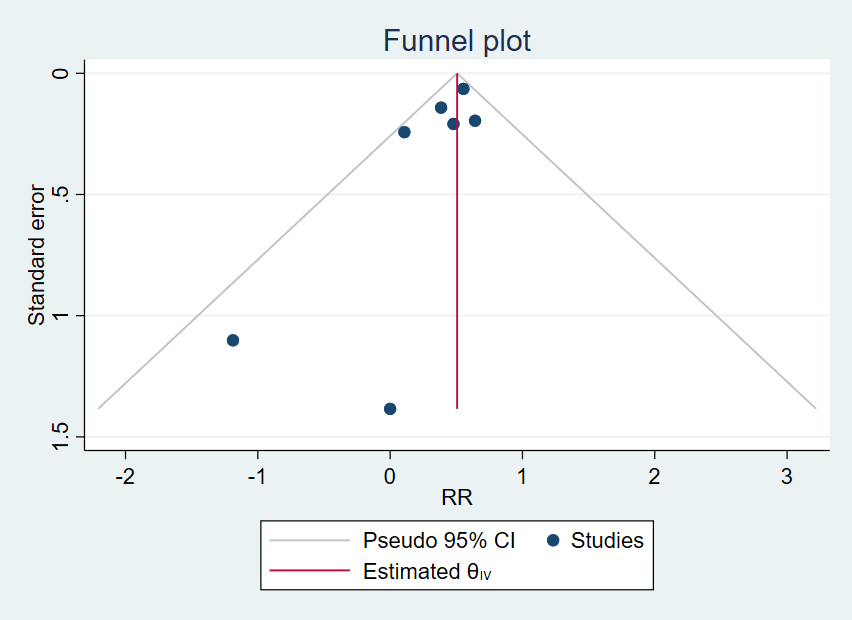

Supplement: Supplementary file 3 — High resolution image (TIF 1549 kb) [file 10389_2021_1654_MOESM2_ESM.tif]
