# Supplementary material for: The risk of non-steroidal anti-inflammatory drug-induced heart failure in people with chronic kidney disease: a systematic review
Source: J Public Health (Berl). 2021 Oct 21;30(7):1763–73. doi: 10.1007/s10389-021-01654-3 (PMC12380885; doi:10.1007/s10389-021-01654-3)
Supplement: Supplementary file 5 — (PDF 119 kb) [file 10389_2021_1654_MOESM4_ESM.pdf]

Supplementary Information Table1: Newcastle Ottawa Scale summary table for quality assessment of observational studies

**Study (Newcastle Ottawa Scale)**	**Selection**	**Comparability**	**Outcome/Exposure**	**Overall**
**Garcia Rodriguez et al. (2003)**	low	low	medium	**low**
**Liu et al. (2016)**	medium	medium	low	**medium**
**Trespalacios et al. (2003)**	low	low	medium	**low**
**Lai et al. (2017)**	medium	low	medium	**medium**
**Kim et al. (2014)**	low	low	medium	**low**
