# Supplementary material for: The risk of non-steroidal anti-inflammatory drug-induced heart failure in people with chronic kidney disease: a systematic review
Source: J Public Health (Berl). 2021 Oct 21;30(7):1763–73. doi: 10.1007/s10389-021-01654-3 (PMC12380885; doi:10.1007/s10389-021-01654-3)
Supplement: Supplementary file 6 — (PDF 200 kb) [file 10389_2021_1654_MOESM5_ESM.pdf]

Supplementary Information Table 2: GRADE pro full assessment

GRADE **pro Evidence Table**

Author(s): Bethany Ward

Question: Aspirin compared to no aspirin in chronic kidney disease

Setting: Primary and/or secondary care

Bibliography: Lai et al. 2017; Liu et al. 2016; Trespalacios et al. 2003; Goicoechea et al. 2018; Sinsakul et al. 2007; and García Rodríguez et al. 2003, Kim et al. 2014

Certainty assessment							№ of patients		Effect		Certainty	Importance
№ of studies	Study design	Risk of bias	Inconsistency	Indirectness	Imprecision	Other considerations	Aspirin	no aspirin	Relative (95% CI)	Absolute (95% CI)		
**Heart Failure (assessed with: NYHA or ICD criteria)**												
3	observational studies	serious	not serious	not serious	not serious	none	392/1182 (33.2%)	234/1344 (17.4%)	**RR 1.43** (1.16 to 1.76)	**75 more per 1,000** (from 28 more to 132 more)	⊕⊕⊕◯ MODERATE	CRITICAL
2	randomised trials	not serious	serious [a]	serious [b]	not serious	none	2/74 (2.7%)	5/85 (5.9%)	not pooled	see comment	⊕⊕◯◯ LOW	
1	observational studies	serious [c]	not serious	serious [d]	not serious	none	490/1380 (35.5%)	279/1371 (20.4%)	**RR 1.74** (1.54 to 1.98)	**151 more per 1,000** (from 110 more to 199 more)	⊕⊕◯◯ LOW	
1	observational studies	serious [e]	not serious	serious [f]	not serious	none	498 cases 1780 controls		**RR 1.9** (1.3 to 2.8)	-	⊕⊕◯◯ LOW	

CI: Confidence interval; **RR:** Risk ratio

Explanations

a. Goicoechea et al. show a significantly reduced risk whereas Sinsakul et al. report no difference in the risks.

b. Indirect due to diabetic nephropathy population and composite endpoint in Sinsakul.

c. Kim et al. found to be prone to some bias in both Newcastle-Ottawa Scale and ROBINS-I due to some presence of outcome at the beginning of the study and undocumented method of outcome measurement.

d. Indirectness in Kim et al. due to composite outcome of cardiovascular event.

e. Garcia Rodriguez was found to have selection bias and some bias due to missing data through the ROBINS-I tool.

f. Garcia Rodriguez use a composite population which only has a small number of renal failure participants, so this is indirect.
